# Artificial sweeteners are related to non-alcoholic fatty liver disease: Microbiota dysbiosis as a novel potential mechanism

**DOI:** 10.17179/excli2020-1226

**Published:** 2020-05-12

**Authors:** Hadi Emamat, Hamid Ghalandari, Hadith Tangestani, Afsoun Abdollahi, Azita Hekmatdoost

**Affiliations:** 1 Student Research Committee, Shahid Beheshti University of Medical Sciences, Tehran, Iran; 2 Department of Clinical Nutrition and Dietetics, Faculty of Nutrition Sciences and Food Technology, National Nutrition and Food Technology Research Institute, Shahid Beheshti University of Medical Sciences, Tehran, Iran; 3 Department of Nutrition, Persian Gulf Tropical Medicine Research Center, Bushehr University of Medical Sciences, Bushehr, Iran; 4 Department of Community Nutrition, School of Nutritional Sciences and Dietetics, Tehran University of Medical Sciences, Tehran, Iran; 5 Department of Nutrition Science, Purdue University, USA

**Keywords:** non-alcoholic fatty liver disease, NAFLD, microbiome, microbiota, artificial sweeteners

## Abstract

Non-alcoholic fatty liver disease (NAFLD) is a systemic and wide-spread disease characterized by accumulation of excess fat in the liver of people who drink little or no alcohol. Artificial sweeteners (ASs) or sugar substitutes are food additives that provide a sweet taste, and are also known as low-calorie or non-calorie sweeteners. Recently people consume increasingly more ASs to reduce their calorie intake. Gut microbiome is a complex ecosystem where 10^14^ microorganisms play several roles in host nutrition, bone mineralization, immune system regulation, xenobiotics metabolism, proliferation of intestinal cells, and protection against pathogens. A disruption in composition of the normal microbiota is known as ‘gut dysbiosis’ which may adversely affect body metabolism. It has recently been suggested that dysbiosis may contribute to the occurrence of NAFLD. The aim of the present study was to investigate the effects of ASs on the risk of NAFLD. The focus of this review is on microbiota changes and dysbiosis. Increasing evidence shows that ASs have a potential role in microbiota alteration and dysbiosis. We speculate that increased consumption of ASs can further raise the prevalence of NAFLD. However, further human studies are needed to determine this relationship definitively.

## Introduction

Non-alcoholic fatty liver disease (NAFLD) is a systemic disease affecting several extra-hepatic organs and regulatory pathways (Byrne and Targher, 2015[8]). This disease consists of a series of events that will eventually lead to accumulation of excess fat in the liver of people who drink little or no alcohol (Chalasani et al., 2018[11]). NAFLD is a wide-spread disease and its current population-based prevalence is approximately 30 % to 40 % in men and 15 % to 20 % in women (Browning et al., 2004[7]). NAFLD is predicted to be the most common cause of liver transplantation by 2030 (Byrne and Targher, 2015[8]). NAFLD increases overall mortality by 57 % and doubles the risk of type 2 diabetes (Musso et al., 2011[34]) and chronic kidney disease (CKD) (Musso et al., 2014[35]). Obesity (Polyzos et al., 2019[37]), visceral ectopic fat accumulation (Ko et al., 2017[25]), adipose tissue inflammation (du Plessis et al., 2015[14]), type 2 diabetes (Bhatt and Smith, 2015[5]), nutrition and dietary pattern (Emamat et al., 2018[17]; Emamat, 2019[16]; George et al., 2018[20]; Mirmiran et al., 2017[31]), and intestinal dysbiosis (Saltzman et al., 2018[41], Kolodziejczyk et al., 2019[26]) are possible factors involved in the initiation and progression of NAFLD. 

Artificial sweeteners (ASs) or sugar substitutes are food additives that provide a sweet taste, and are also known as low-calorie (e.g. sugar alcohols) or non-calorie (e.g. Aspartame, Acesulfame K, Sucralose and etc.) sweeteners (Lohner et al., 2017[28]). The G-proteins coupled receptors of taste on the tongue perceive the sense of sweetness. Sucrose provides a scale by which the sweetness of sweeteners is measured (Shallenberger and Acree, 1971[44]). Given that consumption of sugars, especially sucrose and glucose-fructose syrups, has dramatically increased worldwide with undesirable effects on body metabolic status, individuals consume increasingly more ASs to reduce their calorie intake (Stanhope, 2016[45]). Up to now, six ASs for foods and drinks including acesulfame potassium (acesulfame K), aspartame, neotame, saccharin, sucralose, and advantame have been approved by the FDA (2014[19]). Although the FDA and many national authorities have recognized that ASs are generally safe and well-tolerated, there is controversy about the effects of these sweeteners on human health (Lohner et al., 2017[28]). Ruanpeng et al. showed a significant association between ASs consumption and obesity in a meta-analysis (Ruanpeng et al., 2017[39]). According to a review study by Pearlman et al. in both animal models and humans, ASs may change the host microbiome, leading to decreased satiety, alteration in glucose homeostasis, increased calorie intake, weight gain and metabolic syndrome (Pearlman et al., 2017[36]).

Gut microbiome is a complex ecosystem where 10^14^ microorganisms, mainly including bacteria, virus, and fungi coexist and may play several roles in host nutrition, bone mineralization, immune system regulation, xenobiotics metabolism, proliferation of intestinal cells, and protection against pathogens (Gill et al., 2006[21], Seksik and Landman, 2015[43]). Microbiome, like fingerprint, is individual-specific; nevertheless, several factors such as genetics, diet, antibiotic therapy, and environmental changes can alter it (Faith et al., 2013[18]; Lozupone et al., 2012[29]). A healthy state of the gut microbiota may consist of a lower number of pathogenic species such as *Campylobacter jejuni*, *Salmonella enterica*, *Vibrio cholerae*, and a higher number of non-pathogenic genera, including *Bacteroides*, *Prevotella* and *Ruminococcus* (Hollister et al., 2014[22]). A quantitative or qualitative disruption in composition of the normal microbiota is known as ‘gut dysbiosis’ which may adversely affect body metabolism and immune responses. It has recently been suggested that dysbiosis may contribute to the occurrence of NAFLD (Carding et al., 2015[10]).

Recent findings raise concerns about the negative effects of ASs on health. The aim of the present study is to investigate the effects of ASs on the risk of NAFLD. The focus of this review is on microbiota changes and dysbiosis.

## Dysbiosis and NAFLD

In recent years, studying the relationship between the microbiota and its potential role in the pathogenesis of NAFLD has been a subject of interest (Saltzman et al., 2018[41]; Kolodziejczyk et al., 2019[26]; Duarte et al., 2019[15]). A growing body of evidence suggests that along with diet, physical activity and genetic predisposition, the gut microbiota affects hepatic metabolism as well as the inflammatory status of the liver. Therefore, microbiota may be involved in development of NAFLD and its progression to NASH (Kolodziejczyk et al., 2019[26]; Duarte et al., 2019[15]). In the following sections, we focus on the studies and mechanisms underlying the impact of dysbiosis on NAFLD pathogenesis. Table 1(Tab. 1) summarizes the mechanisms underlying the impact of dysbiosis on NAFLD.

NAFLD is one of the most important comorbidities of obesity. The gut microbiota has an important role in harvesting energy from the diet and can result in adiposity (Turnbaugh et al., 2006[47]). The underlying mechanisms are improved development of the small intestinal epithelium and impact on the gut physiology and motility (Lichtman et al., 1991[27]). Furthermore metagenomic studies suggest that the gut microbiota in obese mice had a higher potential for harvesting energy (Turnbaugh et al., 2006[47]).

Bacterial components activate Toll-like receptors (TLRs) in the gut mucosa. Transferring the gut microbiota of mice affected by metabolic syndrome into the gut of normal mice resulted in the development of metabolic syndrome in normal mice. This observation supports the crucial role of gut microbiota in the development of metabolic syndrome (Rivera et al., 2007[38]). The healthy intestinal epithelium forms a tightly sealed physical barrier. Dysbiosis disrupts the gut epithelial barrier and increases intestinal permeability. A leaky gut leads to the passage of pro-inflammatory molecules and bacterial endotoxins to bloodstream reaching the liver via the portal vein and increases hepatic inflammation and the susceptibility to NAFLD (Muñoz et al., 2019[33], Cani et al., 2007[9]). Lipopolysaccharides (LPS) produced by gram-negative bacteria lead to insulin resistance through TLR4-dependent activation of the NF-κB pathway and increase inflammation in the liver (Boulange et al., 2016[6]; Cani et al., 2007[9]). Another microbiota-derived mediators related to NAFLD are short-chain fatty acids (SCFAs) (Juárez-Hernández et al., 2016[24]). SCFAs including acid acetic acid, propionic acid, and butyric acid are fatty acids with seven or fewer carbon atoms, mainly produced from indigestible carbohydrate fermentation (Chambers et al., 2018[12]). SCFAs are the main energy source for gut epithelial cells, help to preserve the intestinal integrity, and also have several effects on enery metabolism, immune response, and adipose tissue expansion (Arslan, 2014[4]). Dysbiosis results in an increase of SCFAs that can in turn promote hepatic lipogenesis (mainly by acetate), and gluconeogenesis (mainly by propionate) leading to liver steatosis (Morrison and Preston, 2016[32]). Also, SCFAs regulate the production of several inflammatory cytokines, including tumor necrosis factor-α (TNF-α), interleukin-2 (IL-2), IL-6, and IL-10 that are involved in the pathogenesis of NAFLD (Vinolo et al., 2011[49]). Literature provides evidence of SCFAs potential use as indicator of NAFLD progression (Aragonès et al., 2019[3]). Carbohydrate fermentation by gut microbiota has another by-product, i.e. ethanol, which could promote NAFLD (Zhu et al., 2013[51]). Blood ethanol concentrations are higher in NAFLD patients compared to healthy subjects suggesting that endogenous ethanol production may contribute to the liver damages by increasing several inflammatory signals (Zhu et al., 2016[50]). A metabolite of phenylalanine, phenyl acetate, is another microbiota-derived metabolite that is higher in the blood of NASH patients and is associated with disease severity (Hoyles et al., 2018[23]).

Activation or modulation of bile acid receptors, such as the farnesoid X receptor and TGR5, and transporters, such as the ileal apical sodium-dependent bile acid transporters, are involved in the pathogenesis of insulin resistance and NAFLD. Given that gut microbiota can change the bile acid pool and signaling characteristics, this may be another possible mechanism of dysbiosis-induced NAFLD development (Arab et al., 2017[2]). 

The mobilization of liver fat depends on production and transportation of very low density lipoprotein (VLDL). The production of this lipoprotein particle depends on the presence of choline. Choline-free diets are commonly used for NAFLD induction in animals. Absence of choline leads to hepatic fat accumulation that causes oxidative stress and alterations in cytokines and adipokines, as well as slight inflammation and fibrosis in the liver (Al Rajabi et al., 2014[1]; Corbin and Zeisel, 2012[13]). The gut microbiota converts choline to dimethylamine (DMA) and trimethylamine (TMA) which may lead to choline deficiency. Dysbiosis enhances the metabolism of choline to TMA and DMA, thus leading to liver-related consequences (Corbin and Zeisel, 2012[13]).

## Artificial Sweeteners and Dysbiosis

Dysbiosis by definition is “an imbalance in the gut microbial community that is associated with disease” (Messer and Chang, 2018[30]). By that definition, it is safe to say that, despite previous misconceptions, some ASs “unequivocally and irrefutably” disrupt gut microbiota (Schiffman and Nagle, 2019[42]). Nonetheless, various AS formulations may have different effects. Moreover, there are several questions regarding the extent and the nature of what happens after consuming certain ASs.

Ruiz-Ojeda et al. (2019[40]) reviewed the then-existing literature on the impact of some FDA-approved ASs on the gut microbiota. Even though in their review they were able to find some relevant data regarding the effect of sucralose and saccharin on the gut microbiota, they could not do the same for other ASs. Based on their findings and also the search that we have conducted, there is scarce information on the effect of other ASs on gut microbiota. Therefore, we discuss sucralose and saccharin in more details.

Sucralose is one of the most widely-consumed ASs around the world. It is 600 times sweeter than sucrose. Studies suggest that sucralose may cause dysbiosis by decreasing the total number of aerobic and anaerobic species, *bifidobacteria*, *lactobacilli*, *Bacteriodes*, and *Clostridiales* (Ruiz-Ojeda et al., 2019[40]). Another study showed that it could increase *Clostridium* cluster XIVa in mice (Uebanso et al., 2017[48]).

Saccharin, also one of the most globally-used sweeteners, has been investigated for its possible role in dysbiosis. Current data suggest that saccharin might inhibit the growth of six bacterial strains: three *lactobacilli* species and three *E. coli* strains (Ruiz-Ojeda et al., 2019[40]). Another study found that saccharin increases *Bacteriodes* genus and, similar to the previous study, reduces the number of *lactobacilli *(Suez et al., 2015[46]).

It needs to be emphasized that there still is a major shortage of original investigations regarding other FDA-approved ASs. Some of them (e.g. neotame, advantame, and cyclamate) have never been examined. Others (e.g. Acesulfame potassium and aspartame) have only been studied to a limited extent; therefore, their impact on the gut microbiota remains unclear and inconclusive. 

Another important issue is that most of the existing information is animal-based. There is a significant need to examine this possible association in human subjects with various dietary approaches; due to the fact that in human beings there are a lot of factors affecting the gut microbiota, most importantly the dietary pattern. Suez et al. (2015[46]) suggested that by following a large cohort of human subjects, they were able to find associations between consumption of non-nutritional sweeteners (NNSs) and a disrupted microbiota. However, more studies are required to reproduce this finding and confirm a true causal relationship. 

Further studies are required to understand the possible mechanisms by which ASs may alter the composition of the gut microbiota. There have been several ‘assumptions’ though. Suez et al. (2015[46]) proposed that sucralose and saccharin may be metabolized by some genera of bacteria, while remain not metabolizable for the rest. This raised the proposition that the number of the bacteria able to metabolize the consumed AS may rise to the disadvantage of the others. However, at this point, this remains suggestive and needs to be confirmed by further more investigation.

## Conclusion

Dysbiosis is one of the factors with demonstrated contribution to the pathogenesis of NAFLD. Increasing evidence shows that ASs have a potential role in microbiota alteration and dysbiosis. We speculate that increased consumption of ASs can further raise the prevalence of NAFLD. However, further human studies are needed to determine this relationship definitively.

## Acknowledgements

This study is related to the project no. 20181 from the Student Research Committee, Shahid Beheshti University of Medical Sciences (SBMU), Tehran, Iran. We also appreciate the Student Research Committee and Research & Technology Chancellor in SBMU for their financial support of this study.

## Conflict of interest

The authors declared that there is no conflict of interest.

## Figures and Tables

**Table 1:**
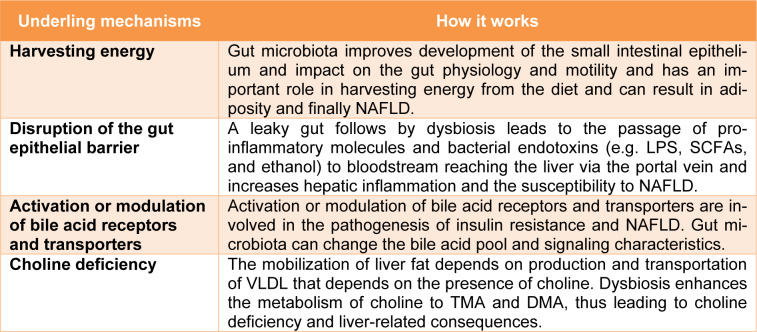
The mechanisms underlying the impact of dysbiosis on NAFLD
